# Convolutional neural network for classification of two-dimensional array images generated from clinical information may support diagnosis of rheumatoid arthritis

**DOI:** 10.1038/s41598-020-62634-3

**Published:** 2020-03-27

**Authors:** Jun Fukae, Masato Isobe, Toshiyuki Hattori, Yuichiro Fujieda, Michihiro Kono, Nobuya Abe, Akemi Kitano, Akihiro Narita, Mihoko Henmi, Fumihiko Sakamoto, Yuko Aoki, Takeya Ito, Akio Mitsuzaki, Megumi Matsuhashi, Masato Shimizu, Kazuhide Tanimura, Kenneth Sutherland, Tamotsu Kamishima, Tatsuya Atsumi, Takao Koike

**Affiliations:** 1 Hokkaido Medical Center for Rheumatic Diseases, Sapporo, Japan; 2 Global Station for Medical Science and Engineering, Global Institution for Collaborative Research and Education, Hokkaido University, Sapporo, Japan; 3 Faculty of Health Sciences, Hokkaido University, Sapporo, Japan; 4 Department of Rheumatology, Endocrinology and Nephrology, Faculty of Medicine and Graduate School of Medicine, Hokkaido University, Sapporo, Japan

**Keywords:** Rheumatoid arthritis, Machine learning, Diagnostic markers

## Abstract

This research aimed to study the application of deep learning to the diagnosis of rheumatoid arthritis (RA). Definite criteria or direct markers for diagnosing RA are lacking. Rheumatologists diagnose RA according to an integrated assessment based on scientific evidence and clinical experience. Our novel idea was to convert various clinical information from patients into simple two-dimensional images and then use them to fine-tune a convolutional neural network (CNN) to classify RA or nonRA. We semi-quantitatively converted each type of clinical information to four coloured square images and arranged them as one image for each patient. One rheumatologist modified each patient’s clinical information to increase learning data. In total, 1037 images (252 RA, 785 nonRA) were used to fine-tune a pretrained CNN with transfer learning. For clinical data (10 RA, 40 nonRA), which were independent of the learning data and were used as testing data, we compared the classification ability of the fine-tuned CNN with that of three expert rheumatologists. Our simple system could potentially support RA diagnosis and therefore might be useful for screening RA in both specialised hospitals and general clinics. This study paves the way to enabling deep learning in the diagnosis of RA.

## Introduction

Rheumatoid arthritis (RA) is a chronic inflammatory disease in which synovitis affects joint structure that then progresses to joint destruction. Recently, molecular targeted therapies such as biological disease-modifying antirheumatic drugs (DMARDs) and targeted synthetic DMARDs have resulted in a revolutionary change in the clinical practice of rheumatology^1^, but several problems still remain. From the diagnostic aspect, definite diagnostic criteria and direct markers for RA are still lacking, and there are many diseases other than RA that also present with joint pain. Rheumatologists use the 2010 American College of Rheumatology (ACR)/The European League Against Rheumatism (EULAR) classification criteria for diagnosis^2^, but many cases do not satisfy the criteria. Patients are diagnosed as having RA when rheumatologists determined that the patients should start antirheumatic therapy even if they do not satisfy the criteria. Rheumatologists make this determination holistically based on various clinical information.

In the present study, various clinical data such as a patient’s joint symptoms, joint tenderness and/or swellings examined by rheumatologist, blood test data and joint ultrasonography were converted into a two-dimensional array (TDA) image for each patient. We aimed to investigate whether a deep learning method based on a convolutional neural network (CNN) could classify the images as RA or nonRA. We used a pre-trained CNN, the AlexNet with transfer learning^3^. In five trials of independent learning, the fine-tuned AlexNet that showed the best accuracy for the testing data was selected, and we then compared the classification of RA determined by the fine-tuned AlexNet with that made by rheumatologists.

## Results

In the five trials of independent learning for the AlexNet, the accuracy identifying the testing data in each learning was 96, 96, 98, 96 and 90%, respectively, with the 3^rd^ fine-tuned AlexNet showed the best accuracy for the testing data. A confusion matrix for the testing data used by the 3^rd^ fine-tuned AlexNet is shown in Table 1. Precision with the 3^rd^ fine-tuned AlexNet was 91% and recall was 100%. The confusion matrix for other classification results (1^st^, 2^nd^, 4^th^ and 5^th^) are shown in Supplemental Tables S1–4. Agreement between the classification determined by the 3^rd^ fine-tuned AlexNet and that made by each of the three rheumatologists was favorable (Cohen’s coefficient kappa = 0.79, 0.83, 0.87, respectively).

**Table 1:** Confusion matrix of the fine-tuned AlexNet for the testing data.

Output labels		RA	nonRA
True labels	RA	10	0
	nonRA	1	39

*Abbreviation, RA = rheumatoid arthritis.

## Discussion

Our study found that CNN classification of TDA images could support the diagnosis of RA.

When expert rheumatologists start anti-rheumatic therapies on patients who do not satisfy the ACR/EULAR classification criteria^2^, they make the decision according to an integrated assessment based on scientific evidence and their own clinical experience. However, it is difficult to convert this expert integrated assessment to a formulaic method. Using traditional statistical method has not succeeded to reproduce the expert integrated assessment. We started to develop algorithm to mimic the integrated assessment of rheumatologists and then came up with the idea to condense clinical information into an image and use these images with artificial intelligence technology. We speculated that image features represent complex disease features of RA. Deep learning is an ongoing progressive technology that is being applied to an expanding number of fields. Image classification based on CNN is one of the most advanced applications among them, and it can sometimes classify various images much better than humans can. Because the CNN method is able to detect image features, we speculate this technology can detect features of TDA images in order to classify RA or nonRA. We focused on this technology and used one of the pre-trained CNNs, the AlexNet, with transfer learning. The AlexNet contains 8 layers (5 convolutional and 3 fully connected layers) and can classify images into 1000 classes^3^. Transfer learning is an application of deep learning in which a pre-trained CNN is used as a base to expand the classification of new images. It is faster and requires fewer learning data than training an initial CNN from the beginning. In our method of generating TDA images, we semi-quantitatively converted each type of clinical information into four coloured square images and arranged them as one image. Mathematically, this process converted one-dimensional data into three-dimensional matrix data. Our idea of converting clinical information into single image was, to our knowledge, novel, unique and without precedent. The AlexNet was fine-tuned with these TDA images using transfer learning.

The main result of the present study was that the CNN of the fine-tuned AlexNet could classify simple TDA images as RA or nonRA with high accuracy. Second, agreement between the classification determined by the AlexNet and that made by the three rheumatologists was favorable. These results indicated that the TDA image actually reflected the clinical condition of RA or nonRA, and the CNN could detect the differences equally as well as expert rheumatologists. We also tested the classification ability of another pre-trained CNN, Resnet-18 for validation^4^. The methods and settings of Resnet-18 are described in Supplemental Information. In the five trials of independent learning for the Resnet-18, the accuracy identifying the testing data in each learning was 94, 90, 96, 98 and 98% (Supplemental Tables S5–9). Both the AlexNet and Resnet-18 classified images with high accuracy. We next focused on images for which two CNNs failed to classify correctly. In the five trials by AlexNet and Resnet-18, the number of false negative images (true = RA, output = nonRA) were 6(/50) and 3(/50), respectively. The number of false positive images (true = nonRA, output = RA) were 3(/200) and 9(/200) for AlexNet and Resnet-18, respectively. Interestingly, there were images which both AlexNet and Resnet-18 failed to classify, two false negative and two false positive; however, other failed images were varied. But because two CNNs showed high accuracy, the concept of our study seems to be valid. However, focusing on the images which the two CNNs failed to classify, classification failure might be varied by different CNNs. The different architecture of the two CNNs might influence feature extraction. This might pose a risk to misdiagnosis. To develop our system to use in actual clinical practice and prevent misdiagnosis, system which consist of several CNNs architectures may be useful.

It was interesting what the AlexNet focused on in the TDA images to classify them. We speculated that it might identify several specific imaging patterns. In the present study, anti-citrullinated protein/peptide antibody (ACPA) was clinically important for diagnosis and was significantly higher in the RA learning data (Table 4) although the fine-tuned AlexNet could correctly judge RA negative for ACPA as RA. Interestingly, our system failed to judge nonRA negative for ACPA when ACPA negativity was experimentally changed to positive in several cases. These results indicated that our system might recognise ACPA as important in the determination of RA and also that the system might judge RA based not only on ACPA but also other variables; thus, it could still determine RA negative for ACPA as being RA. To study more about what the AlexNet focused on in the TDA images, we performed an additional test. The TDA image was separated into two images: one with only cruciform block consist of direct joint information and another image with clinical information such as blood test. We used these images for fine-tuning the AlexNet in the same method as the main test. The results were that the classification accuracy of cruciform block images was 88, 92, 90, 84 and 94% (Supplemental Tables S10–14), while the result with images containing other clinical information was 80, 80, 78, 78, 74% (Supplemental Tables S15–19). The cruciform block images showed a higher accuracy than images of other clinical information; however, both were inferior to the original TDA image. We speculated that the AlexNet focused on the cruciform block more closely than other clinical information. Furthermore, the combination of cruciform block and other clinical information seemed to improve the performance of classification ability. As we mentioned above, because switching the ACPA value influenced the classification output in several cases, we speculate that the AlexNet focused on not only the cruciform block but also other clinical information as a whole. Table 4 shows that other clinical information in the learning data was also significantly different between RA and nonRA. However, the relationships between these variables including patient symptoms and imaging information were unclear and must be complicated. In deep learning, studying the relationships between variables and finding hidden critical variables are important and are an ongoing aspect of research in this area. Further specialised research is needed in the future.

A previous report presented the idea of converting numeric data to images and the possibility of using them in image classification^5^. Our novel idea was to convert various type of clinical data, involving not only numeric but also general examination data, into a simple image. To our knowledge, the present study was the first experimental trial in the medical field. This method could further expand the possibility of studying not only RA but also many other diseases.

There are some limitations in our pilot study. First, joint information was limited to that of the fingers and wrist, and therefore our system could not detect RA in other joints with inflammation. Second, the current design of the TDA image might not be the most appropriate. In the design of TDA image, changing colouring rules such as quantitative transferring, changing block patterns, adding other clinical information which we did not used in the current study are interesting and might be useful for improving classification ability. A new optimal TDA design including information from other joints, other clinical information, new colouring rules and block patterns will be required in the future. Third, a single rheumatologist created the learning data by adding modifications to the original data, and thus the learning data might be strongly influenced by the rheumatologist’s own clinical style and be a source of bias. Multiple rheumatologists should be involved in increasing the amount of learning data to avoid bias. Fourth, the learning data were based on the rheumatologist’s decision of whether to start antirheumatic therapy within two months of the first hospital visit, and therefore our system might be useful in detecting RA patients with active inflammation who needed to start treatment within the period of time. However, our system might be unable to detect patients with unclear low active inflammation who do not need to start treatment immediately even if they have RA. More data may need to be supplied in the future for the CNN to learn various disease conditions of RA. Fifth, the architecture of CNNs that we used in the study was modified by simply replacing the last fully connected layer and classification layer. Modification of the CNN architecture such as using other classifiers is interesting and might improve the classification ability. Study of the optimal CNN architecture with TDA images will be required in the future.

We showed that TDA images generated from clinical information reflected the disease condition and that the CNN could classify the images as indicating RA or nonRA. As an aid to the clinical practice of rheumatology, our system may be useful in the early detection of patients with active RA who need to start the treatment. We are interested in our system being used by general practitioners who were not rheumatologists for screening RA. We also expected that using our system can be used by unexperienced rheumatologists in order to improve their diagnostic ability. Our system may thus contribute to the improvement of the clinical practice of rheumatology.

## Methods

### Patients and clinical examinations

In this study, undiagnosed 249 patients with arthralgia in their fingers or wrists who first visited Hokkaido Medical Center for Rheumatic Diseases during 1 January to 31 May 2019 were enrolled. On the first visit, clinical information was obtained from each patient examined that included location of symptomatic joints with pain and/or stiffness (wrist, 2^nd^ to 5^th^ distal interphalangeal [DIP], 2^nd^ to 5^th^ proximal interphalangeal [PIP], interphalangeal [IP] and 1^st^ to 5^th^ metacarpophalangeal [MCP] joints), body temperature, duration from symptom onset to hospital visit, presence or absence of body trunk pain, skin abnormalities (eruptions, Raynaud phenomenon, etc.) and patient Visual Analog Scale (VAS) score. Rheumatologists (JF, MI, TH, MM, MS, KT, TKo) checked for tenderness and swelling joints and then also recorded the doctor VAS. Blood test for white blood cell count, C-related protein, erythrocyte sedimentation rate, rheumatoid factor, ACPA, anti-nuclear antibody and matrixmetalloproteinase-3 were performed. Patients were diagnosed as having RA when the rheumatologists determined the need to start antirheumatic therapy within 2 months of the first visit. This study was based on actual daily clinical practice, and therefore patients who did not satisfy the 2010 ACR/EULAR classification criteria were also included.

### Joint ultrasonography

Joint ultrasonography of the finger (DIP, PIP, IP, MCP) and wrist joints was performed in each patient by expert sonographers (AN, MH, FS)^6,7^. Synovitis was scored according to the Outcome Measures in Rheumatology (OMERACT) semi-quantitative method in grayscale (0–3) and power Doppler modes (0–3)^8,9^.

### Converting clinical information to two-dimensional array images

**Figure 1:**
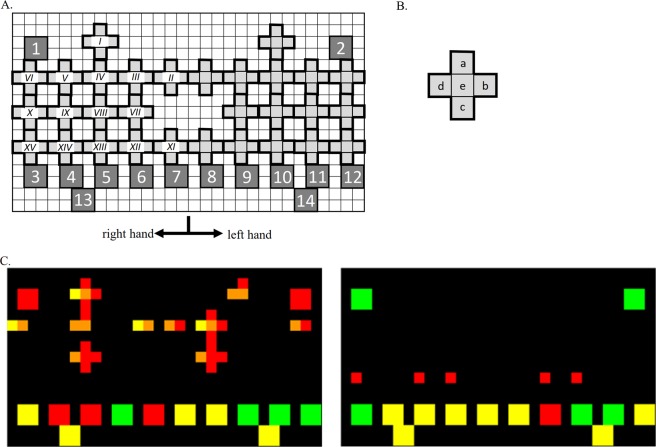
Design of the two-dimensional array image. **(A)** Cruciform blocks indicated each joint (*I*: wrist, *II*: MCP1, *III*: MCP2, *IV*: MCP3, *V*: MCP4, *VI*: MCP5, *VII*: PIP2, *VIII*: PIP3, *IX*: PIP4, *X*: PIP5, *XI*: IP, *XII*: DIP2, *XIII*: DIP3, *XIV*: DIP4, *XV*: DIP5). The array of the cruciform blocks is bilaterally symmetrical. Square blocks with number inside (1–14) show clinical information. Colouring rules for the blocks are listed in Table 2. (**B)** The cruciform block pattern consists of five squares labelled a, b, c, d, and e, with each indicating joint information. Colouring rules for the blocks are also listed in Table 2. (**C**) Representative image of RA (C-1) and that of nonRA (C-2) are shown. RA = rheumatoid arthritis, MCP = metacarpophalangeal, DIP = distal interphalangeal, PIP = proximal interphalangeal, IP = interphalangeal.

The design of the TDA image is shown in Fig. 1A,B and Table 2, and representative TDA images are shown in Fig. 1C. We used Excel software (ver 1909, Microsoft, Redmond, WA) to convert the clinical information into TDA images, which were then saved as tiff files (871 × 494 pixels). Colouring rules for the blocks shown in Table 2 were defined by rheumatologists who were involved in the study (JF, MI, TH, MM, MS, KT, TKo). As it becomes significant, the colour changes green to yellow, orange then red. The cutoff value of each of clinical information shown in Table 2 were also defined by the same rheumatologists (JF, MI, TH, MM, MS, KT, TKo) according to their expert opinions.

**Table 2:** Converting clinical information to two-dimensional array image.

Objects shown in Fig. 1A,B	Clinical information	Colouring rules
**square**		
1	RF (IU/mL)	green < 15, 15~30 yellow, 30 ≤ red
2	ACPA (U/mL)	green < 4.5, 4.5 ≤ red
3	ESR (mm/hour)	green < 10, 10~20 yellow, 20 ≤ red
4	Symptom onset to visit (days)	yellow < 42, 42 ≤ red
5	CRP (mg/dL)	yellow < 0.3, 0.3 ≤ red
6	ANA (n times)	0 = green, 40 or 80 = yellow, 160 ≤ red
7	MMP-3 (ng/mL)	yellow < 120, 121 ≤ red
8	WBC (/uL)	green < 4000, 4000~8000 yellow, 8000 ≤ red
9	gender	male = yellow, female = red
10	skin abnormality	negative = green, positive = yellow
11	body temperature (°C)	green < 37.5, 37.5~38 yellow, 38 ≤ red
12	body trunk pain	negative = green, positive = yellow
13	patient-VAS	green < 10, 10~50 yellow, 50 ≤ red
14	doctor-VAS	green < 10, 10~50 yellow, 50 ≤ red
**cruciform block**		
a	patient joint symptom	negative = green, positive = red
b	doctor examined joint tender	negative = green, positive = red
c	doctor examined joint swelling	negative = green, positive = red
d	ultrasound gray scale score	1 = yellow, 2 = orange, 3 = red
e	ultrasound power Doppler score	1 = yellow, 2 = orange, 3 = red

*Abbreviation, RA = rheumatoid arthritis, RF = rheumatoid factor, ACPA = anti-citrullinated protein/peptide antibody, ESR = erythrocyte sedimentation rate, CRP = C-related protein, ANA = anti-nuclear antibody, MMP-3 = matrixmetalloproteinase-3, WBC = white blood cell count and VAS = visual analog scale.

### Testing data for fine-tuned convolutional neural network and rheumatologists

Testing data from 10 RA patients and 40 nonRA patients were randomly selected and used for estimation of the classification by the fine-tuned CNN and the rheumatologists as mentioned below. Characteristics of the data are shown in Table 3. The 2010 ACR/EULAR classification criteria scores for the 10 RA patients were a score of 1 in one patient, 4 in two, 5 in two, 6 in two, 7 in one and 10 in two patients. Diagnoses of joint pain in the 40 nonRA patients were osteoarthritis in 17 patients, primary Sjögren syndrome in two, fibromyalgia in one, tendonitis in one and no sign of musculoskeletal disease in 19 patients.

**Table 3:** Characteristics of the testing data.

	RA	nonRA	
	(n = 10)	(n = 40)	
RF (IU/mL, median, range)	13 (0–236)	5 (0–313)	*P* = *0.43*
ACPA (U/mL, median, range)	2.4 (0–680)	0 (0–724)	*P* = *0.0013*
ESR (mm/hour, median, range)	26 (6–98)	10 (4–59)	*P* = *0.024*
**Symptoms onset to visit**			
(days, median, quartile range)	150 (56–315)	60 (25–365)	*P* = *0.18*
CRP (mg/dL, median, range)	0.68 (0.06–11.1)	0.035 (0.01–1.67)	*P* < *0.0001*
ANA (n times, median, range)	80 (0–160)	0 (0–1280)	*P* = *0.001*
MMP-3 (ng/mL, median, range)	97.7 (49.1–195)	37.3 (22.2–70.1)	*P* < *0.0006*
WBC (/uL, median, range)	7150 (4900–10800)	6100 (2900–13400)	*P* = *0.0011*
gender (female/male)	6/4	34/6	*P* = *0.08*
skin abnormality (positive/negative)	0/10	1/39	*P* = *0.071*
body temperature (°C, range)	36–37.2	35.8–37	*P* = *0.10*
body trunk pain (positive/negative)	1/9	5/35	*P* = *0.83*
patient-VAS (0–100, median, range)	35 (0–90)	30 (0–80)	*P* = *0.77*
doctor-VAS (0–100, median, range)	22.5 (0–88)	6 (0–70)	*P* = *0.19*

*Abbreviation, RA = rheumatoid arthritis, RF = rheumatoid factor, ACPA = anti-citrullinated protein/peptide antibody, ESR = erythrocyte sedimentation rate, CRP = C-related protein, ANA = anti-nuclear antibody, MMP-3 = matrixmetalloproteinase-3, WBC = white blood cell count and VAS = visual analog scale.

### Learning data for fine-tuning of the convolutional neural network

The data from 42 RA and 157 nonRA patients were used as original learning data after subtracting the data used as testing data. Testing and training data were completely separated. Diagnoses of the 157 nonRA patients were osteoarthritis in 63 patients, systemic lupus erythematosus in three, primary Sjögren syndrome in four, fibromyalgia in three, virus infection in two, tendonitis in two, gout in one, and no sign of musculoskeletal disease in 79 patients. One rheumatologist (JF) added some modifications to the clinical information of each patient to increase the amount of learning data. One data was extracted individually and then increased the number by data modification (RA was increased from one to six and nonRA was increased from one to five). The data modification rule stipulated that the rheumatologist could freely add modifications to clinical information, but they could not affect the original diagnosis. In total, the final number of learning data (original plus artificial data) were 252 for RA and 785 for nonRA. Characteristics of the final learning data are shown in Table 4. In the actual process, the data were randomly split to training (80%) and validation (20%) data and then used for fine-tuning of the CNNs. These processes were independently performed five times, and the CNN showing the best accuracy for the testing data was chosen.

**Table 4:** Characteristics of the final learning data.

	RA	nonRA	
	(n = 252)	(n = 785)	
RF (IU/mL, median, range)	68 (0–2265)	5 (0–810)	*P* < *0.0001*
ACPA (U/mL, median, range)	(0–3519)	0 (0–145)	*P* < *0.0001*
ESR (mm/hour, median, range)	36 (5–111)	10 (5–67)	*P* < *0.0001*
**Symptoms onset to visit**			
(days, median, quartile range)	60 (30–120)	60 (30–360)	*P* = *0.0098*
CRP (mg/dL, median, range)	0.63 (0–14.8)	0.05 (0–5.6)	*P* < *0.0001*
ANA (n times, median, range)	0 (0–640)	0 (0–2560)	*P* = *0.0026*
MMP-3 (ng/mL, median, range)	116 (16–706)	38.3 (10–155)	*P* < *0.0001*
WBC (/uL, median, range)	7200 (2700–12200)	5700 (2100–12800)	*P* < *0.0001*
gender (female/male)	150/102	585/200	*P* < *0.0001*
skin abnormality (positive/negative)	0/252	25/760	*P* = *0.09*
body temperature (°C, range)	35.8–37.4	35.8–37.8	*P* = *0.08*
body trunk pain (positive/negative)	36/216	168/617	*P* = *0.014*
patient-VAS (0–100, median, range)	35 (0–90)	10 (0–90)	*P* < *0.0001*
doctor-VAS (0–100, median, range)	10 (0–80)	1 (0–60)	*P* < *0.0001*

*Abbreviation, RA = rheumatoid arthritis, RF = rheumatoid factor, ACPA = anti-citrullinated protein/peptide antibody, ESR = erythrocyte sedimentation rate, CRP = C-related protein, ANA = anti-nuclear antibody, MMP-3 = matrixmetalloproteinase-3, WBC = white blood cell count and VAS = visual analog scale.

### Fine-tuning of the convolutional neural network

Fine-tuning of one of the pre-trained CNNs, AlexNet, with transfer learning to classify the TDA images was done with the aid of the MATLAB software 2019b (MathWorks, Inc., Natick, MA). The modifications to the architecture of the original pre-trained AlexNet was shown in the following. The 23^rd^ layer of the network architecture was replaced with a fully connected layer that was set to classify two new classes. The final layer was replaced with a classification output layer. Learning options with some modifications as mentioned below were used. The learning rate was slowed down to 0.001 to allow efficient learning new classes. The maximum number of epochs was set as 10, and the validation frequency was set to about once per epoch.

### Agreement for classification between the fine-tuned AlexNet and rheumatologists

Three expert rheumatologists (YF, MK, NA) classified the testing data. We compared the classification determined by the fine-tuned AlexNet with that made by the expert rheumatologists.

### Statistics analysis

Differences in parameters were examined using a nonparametric Mann-Whitney U test. Differences in the ratio between two groups were examined using the chi-squared test. Agreement in the classification by the AlexNet with that by the expert rheumatologists was analysed according to Cohen’s coefficient kappa value, which approaches 1 as concordance becomes stronger (>0.8 is considered to be very good). A value of *P* < *0.05* was considered to indicate statistical significance. Statistical analyses were performed with the use of MedCalc 19.1 (MedCalc Software, Mariakerke, Belgium).

### Ethical considerations

This retrospective study was approved by the local ethics committees of Hokkaido Medical Center for Rheumatic Diseases and Hokkaido University). The study outline was published on the hospital’s homepage at http://www.ra-hp.jp/clinical_research.html, and an opt-out management strategy was used to collect the patient information. All the patients included in the study were above 18 years of age and informed consent was obtained in the form of opt-out on the website. This study was conducted in accordance with the Declaration of Helsinki.

## Supplementary information

Supplementary Information.
