# Supplementary material for: Convolutional neural network for classification of two-dimensional array images generated from clinical information may support diagnosis of rheumatoid arthritis
Source: Sci Rep. 2020 Mar 27;10:5648. doi: 10.1038/s41598-020-62634-3 (PMC7101306; doi:10.1038/s41598-020-62634-3)
Supplement: Supplementary file 1 — Supplementary Information. [file 41598_2020_62634_MOESM1_ESM.pdf]

Convolutional neural network for classification of two-dimensional array images generated from clinical information may support diagnosis of rheumatoid arthritis

Jun Fukae*, MD, PhD[1], Masato Isobe, MD, PhD[1], Toshiyuki Hattori, MD[1], Yuichiro Fujieda, MD, PhD[4], Michihiro Kono, MD, PhD[4], Nobuya Abe, MD[4], Akemi Kitano, MT[1], Akihiro Narita, MT[1], Mihoko Henmi, MT[1], Fumihiko Sakamoto, MT[1], Yuko Aoki, MT[1], Takeya Ito, MD[1], Akio Mitsuzaki, MD[1], Megumi Matsuhashi, MD[1], Masato Shimizu, MD[1], Kazuhide Tanimura, MD[1], Kenneth Sutherland, PhD[2], Tamotsu Kamishima, MD, PhD[3], Tatsuya Atsumi, MD, PhD[4], Takao Koike, MD, PhD[1]

[1]Hokkaido Medical Center for Rheumatic Diseases, Sapporo, Japan

[2]Global Station for Medical Science and Engineering, Global Institution for Collaborative Research and Education, Hokkaido University, Sapporo, Japan

[3]Faculty of Health Science, Hokkaido University, Sapporo, Japan

[4]Department of Rheumatology, Endocrinology and Nephrology, Faculty of Medicine and Graduate School of Medicine, Hokkaido University, Sapporo, Japan

Supplemental Information

Supplemental Table S1.

Confusion matrix of the fine-tuned AlexNet for the testing data

Output labels		RA	nonRA
True labels	RA	9	1
	nonRA	1	39

*abbreviation, RA = rheumatoid arthritis

Supplemental Table S2.

Confusion matrix of the fine-tuned AlexNet for the testing data

Output labels		RA	nonRA
True labels	RA	9	1
	nonRA	1	39

*abbreviation, RA = rheumatoid arthritis

Supplemental Table S3.

Confusion matrix of the fine-tuned AlexNet for the testing data

Output labels		RA	nonRA
True labels	RA	10	0
	nonRA	2	38

*abbreviation, RA = rheumatoid arthritis

Supplemental Table S4.

Confusion matrix of the fine-tuned AlexNet for the testing data

Output labels		RA	nonRA
True labels	RA	6	4
	nonRA	1	39

*abbreviation, RA = rheumatoid arthritis

Supplemental Method

Fine-tuning of the convolutional neural network (Resnet-18).

The modifications to the architecture of the original pre-trained Resnet-18 was shown in the following. The last fully connected layer of the original network architecture was replaced with a fully connected layer that was set to classify two new classes. The final layer was replaced with a classification output layer. Learning options with some modifications as mentioned below were used. The learning rate was slowed down to 0.0001 to allow efficient learning new classes. The maximum number of epochs was set as 8, and the validation frequency was set as 5.

Supplemental Table S5.

Confusion matrix of the fine-tuned Resnet-18 for the testing data

Output labels		RA	nonRA
True labels	RA	8	2
	nonRA	1	39

*abbreviation, RA = rheumatoid arthritis

Supplemental Table S6.

Confusion matrix of the fine-tuned Resnet-18 for the testing data

Output labels		RA	nonRA
True labels	RA	10	0
	nonRA	5	35

*abbreviation, RA = rheumatoid arthritis

Supplemental Table S7.

Confusion matrix of the fine-tuned Resnet-18 for the testing data

Output labels		RA	nonRA
True labels	RA	9	1
	nonRA	1	39

*abbreviation, RA = rheumatoid arthritis

Supplemental Table S8.

Confusion matrix of the fine-tuned Resnet-18 for the testing data

Output labels		RA	nonRA
True labels	RA	10	0
	nonRA	1	36

*abbreviation, RA = rheumatoid arthritis

Supplemental Table S9.

Confusion matrix of the fine-tuned Resnet-18 for the testing data

Output labels		RA	nonRA
True labels	RA	10	0
	nonRA	1	39

*abbreviation, RA = rheumatoid arthritis

Supplemental Table S10.

Confusion matrix of the fine-tuned AlexNet for the testing data (image of cruciform block)

Output labels		RA	nonRA
True labels	RA	9	1
	nonRA	5	35

*abbreviation, RA = rheumatoid arthritis

Supplemental Table S11.

Confusion matrix of the fine-tuned AlexNet for the testing data (image of cruciform block)

Output labels		RA	nonRA
True labels	RA	10	0
	nonRA	4	36

*abbreviation, RA = rheumatoid arthritis

Supplemental Table S12.

Confusion matrix of the fine-tuned AlexNet for the testing data (image of cruciform block)

Output labels		RA	nonRA
True labels	RA	9	1
	nonRA	4	36

*abbreviation, RA = rheumatoid arthritis

Supplemental Table S13.

Confusion matrix of the fine-tuned AlexNet for the testing data (image of cruciform block)

Output labels		RA	nonRA
True labels	RA	8	1
	nonRA	7	33

*abbreviation, RA = rheumatoid arthritis

Supplemental Table S14.

Confusion matrix of the fine-tuned AlexNet for the testing data (image of cruciform block)

Output labels		RA	nonRA
True labels	RA	9	1
	nonRA	2	38

*abbreviation, RA = rheumatoid arthritis

Supplemental Table S15.

Confusion matrix of the fine-tuned AlexNet for the testing data (image excluded cruciform block)

Output labels		RA	nonRA
True labels	RA	5	5
	nonRA	5	35

*abbreviation, RA = rheumatoid arthritis

Supplemental Table S16.

Confusion matrix of the fine-tuned AlexNet for the testing data (image excluded cruciform block)

Output labels		RA	nonRA
True labels	RA	5	5
	nonRA	5	35

*abbreviation, RA = rheumatoid arthritis

Supplemental Table S17.

Confusion matrix of the fine-tuned AlexNet for the testing data (image excluded cruciform block)

Output labels		RA	nonRA
True labels	RA	5	5
	nonRA	6	34

*abbreviation, RA = rheumatoid arthritis

Supplemental Table S18.

Confusion matrix of the fine-tuned AlexNet for the testing data (image excluded cruciform block)

Output labels		RA	nonRA
True labels	RA	5	5
	nonRA	6	34

*abbreviation, RA = rheumatoid arthritis

Supplemental Table S19.

Confusion matrix of the fine-tuned AlexNet for the testing data (image excluded cruciform block)

Output labels		RA	nonRA
True labels	RA	5	5
	nonRA	8	32

*abbreviation, RA = rheumatoid arthritis
